# Early appropriate diagnostics and treatment of MDR Gram-negative infections

**DOI:** 10.1093/jacamr/dlac089

**Published:** 2022-09-13

**Authors:** Matteo Bassetti, Souha S Kanj, Pattarachai Kiratisin, Camilla Rodrigues, David Van Duin, María Virginia Villegas, Yunsong Yu

**Affiliations:** Department of Health Science, University of Genoa, Italy; Infectious Diseases Clinic, Ospedale Policlinico San Martino Hospital – IRCCS, Genoa, Italy; Division of Infectious Diseases, American University of Beirut Medical Center, Beirut, Lebanon; Department of Microbiology, Faculty of Medicine Siriraj Hospital, Mahidol University, Bangkok, Thailand; Department of Microbiology, P. D. Hinduja Hospital and Medical Research Centre, Mumbai, Maharashtra, India; Department of Medicine, University of North Carolina School of Medicine, Chapel Hill, NC, USA; Grupo de Investigaciones en Resistencia Antimicrobiana y Epidemiología Hospitalaria (RAEH), Universidad El Bosque, Bogotá DC, Colombia; Department of Infectious Diseases, Sir Run Run Shaw Hospital, Zhejiang University School of Medicine, Hangzhou, Zhejiang, China; Key Laboratory of Microbial Technology and Bioinformatics of Zhejiang Province, Hangzhou, Zhejiang, China

## Abstract

The term difficult-to-treat resistance has been recently coined to identify Gram-negative bacteria exhibiting resistance to all fluoroquinolones and all β-lactam categories, including carbapenems. Such bacteria are posing serious challenges to clinicians trying to identify the best therapeutic option for any given patient. Delayed appropriate therapy has been associated with worse outcomes including increase in length of stay, increase in total in-hospital costs and ∼20% increase in the risk of in-hospital mortality. In addition, time to appropriate antibiotic therapy has been shown to be an independent predictor of 30 day mortality in patients with resistant organisms. Improving and anticipating aetiological diagnosis through optimizing not only the identification of phenotypic resistance to antibiotic classes/agents, but also the identification of specific resistance mechanisms, would have a major impact on reducing the frequency and duration of inappropriate early antibiotic therapy. In light of these considerations, the present paper reviews the increasing need for rapid diagnosis of bacterial infections and efficient laboratory workflows to confirm diagnoses and facilitate prompt de-escalation to targeted therapy, in line with antimicrobial stewardship principles. Rapid diagnostic tests currently available and future perspectives for their use are discussed. Early appropriate diagnostics and treatment of MDR Gram-negative infections require a multidisciplinary approach that includes multiple different diagnostic methods and further consensus of algorithms, protocols and guidelines to select the optimal antibiotic therapy.

## Introduction

In the USA, resistant bacteria and fungi have been estimated to cause at least 2 868 700 infections annually, with 35 900 related deaths.^1^ Ten out of the 18 antibiotic-resistant threats identified by the CDC are represented by antibiotic-resistant Gram-negative bacteria (GNB). The term difficult-to-treat resistance (DTR) has been recently coined to identify those GNB exhibiting resistance to all fluoroquinolones and all β-lactam categories, including carbapenems.^2^ Carbapenem-resistant Enterobacterales (CRE, in particular *Klebsiella pneumoniae* carbapenemases [KPC], metallo-β-lactamases [MBL] and oxacillinase [OXA]-type carbapenemases), carbapenem-resistant *Pseudomonas aeruginosa* (CRPA), carbapenem-resistant *Acinetobacter baumannii* (CRAB) and third-generation cephalosporin-resistant GNB are posing serious challenges to clinicians aiming to identify the best therapeutic option for any given patient. Infections due to these organisms have been associated with unfavourable impacts on hospital length of stay (LOS), clinical cure and patients’ survival in several studies.^2–14^

The contribution of the varied antibiotic resistance mechanisms in GNB on the negative outcomes of patients is likely multifactorial and complex. These resistance mechanisms impact empirical or early appropriate antibiotic therapy, and frequently lead to delays in the administration of appropriate antimicrobials.^3–13^ Delayed appropriate therapy, defined by Bonine *et al*.^4^ as no receipt of antibiotic(s) with relevant microbiological activity on or within 2 days of index date, was associated with worse outcomes including increase in LOS, increase in total in-hospital costs and ∼20% increase in the risk of in-hospital mortality/discharge to hospice, regardless of susceptibility status. In addition, time to appropriate antibiotic therapy has been shown as an independent predictor of 30 day mortality in patients with KPC-producing *K. pneumoniae* (Kp) bloodstream infection (BSI), and appropriate antibiotic therapy is recommended to begin within 24 h from the collection of blood cultures.^5^ Although delayed appropriate therapy is a more important driver of outcomes than CRE, the two factors are recognized to be somewhat synergistic.^15^

To reduce the frequency and duration of inappropriate early antibiotic therapy, this must be addressed from different angles: (i) recognizing the patient-level risk of infections due to DTR-GNB based on medical history and previous colonization or infection with resistant organisms; (ii) updating knowledge of the local antimicrobial resistance epidemiology in order to quantify the hospital-level or ward-level risk of DTR-GNB; (iii) improving and anticipating aetiological diagnosis through improving not only identification of phenotypic resistance to antibiotic classes/agents, but also identification of specific resistance mechanisms in view of the availability of novel β-lactam/β-lactamase inhibitors (BL/BLIs) with differential activity against carbapenem-resistant GNBs that produce different types of carbapenemases; and (iv) ensuring rapid de-escalation to targeted therapy after aetiological diagnosis is confirmed in critically ill patients with suspected DTR-GNB infection who initially required broad-spectrum empirical therapy.

**Figure 1.**
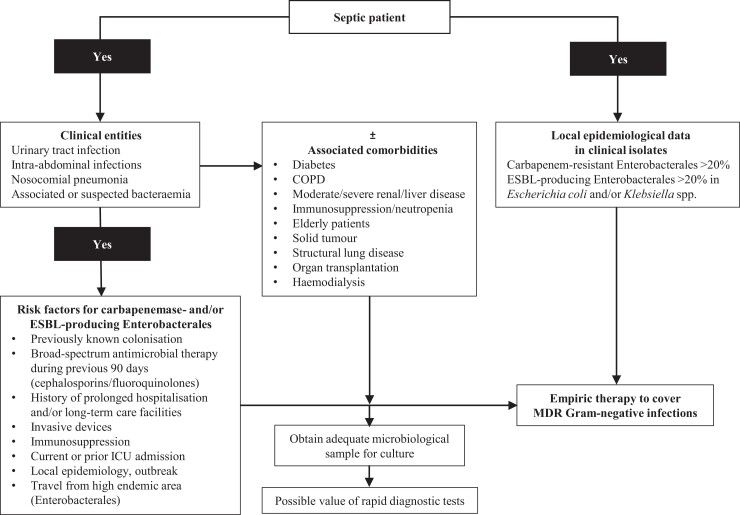
Factors impacting early clinical decision-making when managing MDR Gram-negative bacterial infections. Adapted with permission from: Montravers P, Bassetti M. The ideal patient profile for new β-lactam/β-lactamase inhibitors. *Curr Opin Infect Dis* 2018; **31**: 587–93.^19^

An accurate recognition of the risk of DTR-GNB infection at the patient and hospital levels when starting an empirical antimicrobial treatment is necessary to ensure early appropriate therapy in patients who truly have DTR-GNB infections. It is also necessary to reduce indiscriminate use of antibiotics that are active against DTR-GNB in patients who ultimately do not have such infections. Reducing indiscriminate use helps delay the selection of resistance and reduces the risk of fungal and *Clostridioides difficile* infections.^2,3,8,14,16–18^ Figure 1 shows some of the factors that should be taken into consideration when selecting empirical therapy in patients with potential MDR Gram-negative infections. Regarding points (iii) and (iv) above, the role of the microbiology laboratory is of paramount importance for antimicrobial stewardship (AMS). Rapid diagnostic tests (RDTs) have been combined with AMS interventions, especially in patients with BSIs, showing better clinical outcomes in an increasing number of studies compared with standard methods, as well as having advantages in cost–benefit assessments. However, there remain some challenges in the generalization of results of single studies to other settings (e.g. to hospital/wards with different epidemiology or prevalence of specific pathogens).^6,8,18,20,21^

In light of the above considerations, the present paper reviews the increasing need for rapid diagnosis of bacterial infections and efficient laboratory workflows to confirm diagnoses and allow prompt de-escalation in line with AMS principles.

## RDTs and the role of the clinical laboratory

RDTs, including point-of-care tests (POCTs) and molecular (genotyping) assays, have several advantages compared with standard cultures.^6^ They have higher sensitivity and specificity and accelerate the detection of causative organisms to guide directed therapy. RDTs can be used for both pathogen identification and antimicrobial susceptibility testing (AST), and aid in monitoring response to therapy.^21,22^ In some bacteria, such as CREs, the identification of the molecular mechanism of resistance, e.g. KPC, could inform the early use of new BL/BLIs (e.g. ceftazidime/avibactam, meropenem/vaborbactam and imipenem/relebactam) and potentially better outcomes. One study showed that early use of ceftazidime/avibactam (receipt within 48 h of infection onset) was associated with improved clinical outcomes in patients with MDR Gram-negative infections.^23^ Therefore, the use of RDTs to identify molecular mechanisms of resistance may not only inform the selection of the right antibiotic, including BL/BLI, but will also allow appropriate therapy to be given more quickly. In addition, there are instances where phenotypic breakpoints falter, such as the differential breakpoints for meropenem and meropenem–vaborbactam that can be obtained for OXA-48-producers, further illustrating the importance of genotypic identification.^24^

RDTs have shown promising benefits, especially when coupled with AMS programmes; in particular, reducing time to pathogen identification, which was defined by the time elapsing from collection of specimens to the identification of the causative organisms.^25–28^ Their use can guide clinicians in promptly optimizing antibiotic choice with ideal pharmacokinetic/pharmacodynamic properties.^25–28^

RDTs are essential in the implementation of AMS efforts as they also allow rapid de-escalation of broad-spectrum antimicrobial agents and reduce the potential emergence of future resistance, as well as achieving reductions in cost.^25,27^ Such interventions have been correlated with better patient outcomes. Perez *et al*.^26^ demonstrated that the use of RDTs reduced all-cause 30 day mortality. In addition, the use of RDTs was associated with a decrease in the hospitalization duration and the LOS in the ICU. In this study, it was found that this correlated with significant reduction in the mean annual hospital costs for each inpatient survivor. All these benefits are best demonstrated in the use of RDTs in BSIs.^25,29^ RDTs for respiratory, CNS and gastrointestinal illnesses have also shown significant promise, although more outcome studies are needed to evaluate their full impact.^25^ RDTs can have an added benefit beyond patient care, including epidemiologic surveillance, and facilitate, in conjunction with standard AST methods, the identification of potential candidates for enrolment in clinical trials of novel treatments for MDR Gram-negative bacterial infections.

## Phenotypic and molecular diagnostic tests that reduce time for reporting antimicrobial susceptibility

Culture methods for determining antimicrobial resistance in GNB remain the gold-standard approach but these are time-consuming, taking 24–48 h to complete, and can delay the initiation of appropriate treatment in acutely ill patients. Such delays may increase the severity of illness and the mortality of the patients.^26,30–32^ Consequently, there is an urgent need for rapid methods for determining resistance profiles in both bacterial cultures and directly in patients’ samples.^33–37^ At present, RDT resistance typing methods tend to be concentrated in hospitals, particularly in ICUs, mainly in the USA and Europe, but access for outpatients and general access in developing countries is currently limited.^6,35,38–40^ Making such diagnostic tools available to GPs and patients in the community, and in all regions worldwide, would create a paradigm shift from empirical to evidence-based treatments of infectious diseases.^22,40,41^ However, due to the possibility of false positives and their impact on interpretation of some of the available tests, other evidence (e.g. standard AST, radiographic, serum biomarker data), in addition to clinical evaluation, may be required to support clinical decision-making.^42^

A range of rapid methods for GNB resistance typing are now available and others are under development (see Table 1). These methods can be divided into two classes: (i) those that detect compounds indicating bacterial growth or degradation of the antibiotic, and (ii) genetic/molecular methods that detect nucleic acid sequences indicative of resistance genes and their expression. The turnaround time of these methods is mostly in the range of 1–8 h, which is substantially shorter than traditional culture methods.^33,38,55,57,60^

**Table 1.** Current rapid methods/methods under development for Gram-negative resistance typing

	Targeted resistance mechanism	Targeted population include	Commercial systems include	Typical turnaround time	Performance	Limitations include
MALDI-TOF^31,32,43–46^	All antibiotics. Genotypic equivalent detects either specific protein fragmentation peak patterns (compared with a data library) or biochemical equivalent detects hydrolysis of antibiotics, uptake of stable isotopes in presence of antibiotic or bacterial growth in presence/absence of antibiotics with internal standard compound	All patients with serious/life-threatening infections/sepsis requiring urgent treatment including BSIs, meningitis	MALDI-TOF resistance typing (Bruker Biotyper^®^ and VITEK^®^ MS)	1–4 h^a^ (with some methods this is dependent on incubation time with antibiotic)	Sensitivity: 80%–100%	High upfront costs due to sophisticated hardware and complex databases/data processing involved.^22,32^
					Specificity: 90%–100% depending on bacterial species and resistance type	
					MALDI-TOF can be more effective as part of an AMS programme	Further optimization is needed for detection of resistance amongst various pathogens^32^ and for differentiation of certain bacterial species.^45^ Continuous upgrades to the databases and optimal sample enrichment will increase its accuracy^45^
Colorimetric tests^22,31,40,47,48^	All antibiotics—susceptibility/resistance detected by multiple different means. Colorimetric approaches include phenotyping, detection of bacterial growth (volatile compounds), degradation of the antibiotic and detection of specific resistance genes	All patients with serious or less serious infections	VITEK^®^ 2XL, BD Phoenix^™^, Beckman Microscan, Sensititre^™^ Aris^™^ 2X	Methods requiring bacterial growth (minimum 12 h, can be as long as 30–40 h in total); nucleic acid amplification methods are more rapid	Sensitivity: 95%	Some colorimetric tests require culture and can be slow.^48^ Also, some colorimetric tests have a narrow antibiotic range and limited panel capacity, can be expensive, and require at least ∼10^5^ cells^22^
					Specificity: 98%–100% (nucleic acid methods can be more variable)	
FISH^34,49–52^	Selected antibiotic resistance—mostly involving ribosomal changes, e.g. clarithromycin and linezolid. FISH has been used for the detection of ESBLs. Frequently used for *Helicobacter* and *Campylobacter* species	Patients with various infections such as gastrointestinal, BSIs and respiratory tract infections		60–90 min—some methods are faster	Variable—sensitivity usually reported to be 80%–100% depending on bacterial species (≥90% sensitivity and >98% specificity reported for *K. pneumoniae* and *P. aeruginosa*)	Requires great skill and experience, can have low sensitivity compared with PCR, the organism(s) causing the infection have to be anticipated before the probes are chosen (requires a structured diagnostic algorithm), the density of pathogens should be ≥100 000 cfu/mL^51^ and FISH probe panels need to be tailored to individual needs^52^
FISH + time-lapse and automated photography^49,53,54^	Time-lapse photography over seconds after photobleaching transiently reveals the presence of certain bacterial species after hybridization with nucleic acid mimics. Used in detecting bacteria and their antibiotic resistance genes in patient samples, e.g. gut mucus	Patients with specific infections such as *Helicobacter pylori*		30–90 min (mostly hybridization time)	Not specified in reports identified	The ability of nucleic acid probes to hybridize efficiently can be hindered by the presence of mucus in the samples^54^
Molecular detection systems (nucleic acid amplification-based)^30,55,56^	Multiple different antibiotic resistance mechanisms including DNA gyrase, ribosomal and PBP mutations, BLs, ESBLs, carbapenemases, and membrane pump and permeability/porin-related resistance	Rapid detection of colonized patients and healthcare workers	GeneXpert, (Cepheid—with different kits e.g. Xpert® Carba-R), Check-MDR (Check-Points), BD MAX^™^ (Becton Dickinson)	1–3 h	Sensitivity: 73%–100%	PCR requires a high copy number of the target gene.^30^ Certain tests (e.g. POC mPCR) can have a high rate of complete or partial test failures, leading to non-concordant results in up to 45% of cases^57^
					Specificity: 90.5%–94.5% (for carbapenemases)	False-positive results can arise from residual DNA from dead bacteria, or by detecting bacteria harbouring (but not expressing) certain genes^31^
DNA microarray^49^	Based on hybridization to detect resistant bacteria in samples such as blood and respiratory specimens. Uses an array of gene sequences for multiple antimicrobial resistance markers, including those for BLs and ESBLs	Patients with various infections	Verigene^®^ Nanosphere, SeptiCyte^®^, VAPChip	2.5–8 h	Sensitivity: 72.9%	Microarrays are still considered too complex and protracted for routine use in the clinic, and the range of organisms that can be detected is limited
					Specificity: 99.1% (for VAPChip assay only)	They are also subject to risk of contamination and are expensive to run^49^
NG-Test^®^ CARBA 5 immunochromatographic test^58,59^	The qualitative test CARBA 5 will detect the five most common carbapenemase families (KPC, OXA-48-like, VIM, IMP and NDM) directly from Enterobacterales and *P. aeruginosa* bacterial colonies	For use as an infection control aid in the detection of carbapenemase-producers in healthcare settings		≤15 min^a^	Comparison with composite reference method:	Requires overnight culture, it has only been validated with certain types of agar and with Enterobacterales and *P. aeruginosa*. Organism identification is required prior to testing, and further validation across different sample types (e.g. blood and urine) is needed^58,59^
					PPA: 98.9%–100%	
					NPA: 95.2%–100%	
Clinical metagenomics^42^	NGS of nucleic acids isolated from clinical samples is performed to detect all microbes simultaneously	Intended for rapid and unbiased pathogen identification in clinical specimens	Nanopore sequencing platform (Oxford Nanopore)	6–8 h	Sensitivity: 96.6%	False positives are possible, which may need additional radiographic/clinical investigation
					Specificity: 88.0%	
					PPV: 92.3%	Incurs much greater costs than traditional methods^42^
					NPV: 94.5% (sputum and BAL samples)	

BL, β-lactamase; FISH, fluorescence in-situ hybridization; NPA, negative percentage agreement; POC, point-of-care; PPA, positive percentage agreement; PPV, positive predictive value.

a Does not include the time required if an initial bacterial culture is needed before sample processing.

Proteomics is a technological innovation that has become an integral part of clinical microbiology, with MALDI-TOF MS used for accurate and rapid organism identification.^43^ MALDI-TOF can be complemented with automated phenotypic tests,^33^ and used in conjunction with various molecular platforms to detect specific genes associated with resistance.^18^ The syndromic approach that covers pathogens responsible for clinical presentations such as BSIs, and the most relevant resistance determinants, is revolutionary and enables physicians to make timely clinical decisions.^6,33,61^

In addition, phenotypic AST is universally applicable, mechanism-independent and has therapeutic relevance. This includes tests for detection of carbapenemases including SuperCARBA medium, CHROMID^®^ CARBA SMART, triple disc diffusion using meropenem discs supplemented with aminophenylboronic acid, dipicolinic acid and cloxacillin for KPC, MBL and AmpC detection, respectively, and a temocillin disc zone <10 mm for OXA-48 detection.^62–67^ Other tests include the CARBA NP test or RAPIDEC^®^ CARBA NP test and modified carbapenem inactivation method (mCIM) and, more recently, the immunochromatographic tests such as NG-Test^®^ CARBA 5 and miniaturized magnetic resonance technology; the T2 Biosciences^®^ T2Resistance^®^ Panel can detect resistance genes for the following: KPC, OXA-48, New Delhi MBL (NDM), Verona integron-encoded MBL (VIM), imipenemase (IMP), cefotaximases (CTX-M-14/15) and AmpC.^58,64,68,69^ Some molecular antimicrobial susceptibility tests offer detection of resistance genes in as little as 15 min to 1 h, but these are limited to the most common genes, and negative results do not necessarily imply that the organism is completely susceptible. There are several FDA-approved assays that detect selected resistance genes, including Xpert^®^ Carba-R (Cepheid), the BioFire^®^ Blood Culture Identification 2 (BCID2) Panel (bioMérieux) and the Verigene^®^ system.^70–73^

Despite the availability of many different AST methods, the use of new antibiotics can be hampered by their absence from panels in automated systems and ambiguous verification requirements with existing AST methods at clinical laboratories.^74^ This can place an unnecessary burden on laboratories and may not be possible in smaller centres. To accelerate access to new antibiotics, it has been suggested that laboratories should not need to perform additional verification studies if the AST method to be used is already established and laboratories should not delay using AST for new drugs if recommended quality control testing can be used in parallel.^74^

Data on the proportions of laboratories that have implemented RDTs for GNB resistance typing are not available and widespread use is limited by several barriers. These include the utility of new methods, validation of new technology against reference methods, legal and regulatory landscapes, costs of equipment/funding, costs of maintenance and supplies, reagent stability, optimization of target product profiles, staff experience/training, evaluation and quality control issues.^44,75^ Some methods such as MALDI-TOF have high upfront costs for the equipment but lower running costs; whereas, peptide nucleic acid-fluorescence *in-situ* hybridization (PNA-FISH) and spectrophotometric assays have lower equipment costs but greater running costs in terms of reagents and supplies. The ability to deliver faster resistance results has been shown to reduce antibiotic use, other treatment needs and hospital costs.^8,26,76^

## The impact of RDTs on patient outcomes in healthcare-associated pneumonias, BSIs and complicated urinary tract infections (cUTIs)

A good example of the impact of RDTs on healthcare-associated infections is the integration of novel and rapid diagnostics for resistance phenotyping in patients with hospital- and ventilator-acquired pneumonia (HAP and VAP) as it can potentially significantly improve outcomes.^49,77^ HAP is still a serious infection and an important cause of morbidity and mortality.^49^ In one study, the respiFISH^®^ HAP Gram (−) Panel using fluorescence-DNA molecular beacons (Miacom Diagnostics) shortened identification time by 1 working day (species-level identification within 30 min) with sensitivity and specificity of 94.3% and 87.3%, respectively, relative to standard culture methods.^50^ In this study, 3.6% of pathogens were not identified and 3% of specimens had false-positive results.^50^ However, an observational study examining multiplex PCR (mPCR) (Unyvero, Curetis AG) in 40 patients with HAP reported shortened turnaround times, but complete test failure was seen in 10% of patients (*n *= 4) and partial test failure in 30% (*n *= 12).^57^ There were non-concordant results in 45% of patients (*n *= 18), although concordance improved in a subgroup with more serious pulmonary infections. Whilst this performance was poor, the system may be improved to decrease failure and improve concordance with traditional methods.^57^

Advances in clinical metagenomics using next-generation sequencing (NGS) could offer shorter turnaround times for pathogen identification, and potentially detect all pathogens in a nucleic acid sample simultaneously—a benefit over PCR techniques.^42^ A prospective, single-centre study, which included 66 patients with HAP and analysed sputum and bronchoalveolar lavage (BAL) samples, found a turnaround time of 6.4 ± 1.4 h with a commercial rapid metagenomics test (Simcere Diagnostics) and a sensitivity of 96.6% and specificity of 88.0%.^42^ Due to the high costs of NGS tests, however, it is unlikely that this emerging technology will be used over conventional tests in the immediate future.^42^

Resistant GNB, especially those producing ESBLs and carbapenemases, are an increasingly important aetiology of VAP. RDTs are urgently needed in the management of VAP to facilitate more targeted and appropriate early therapy.^78^ The value of rapid genotypic methods using specific PCR amplification of resistance genes was demonstrated in a study of 66 ESBL isolates from patients with VAP (28 *K. pneumoniae*, 38 *Escherichia coli*).^79^ Among these, the PCR method appeared to identify ESBLs with 100% sensitivity and specificity, and was superior to phenotypic methods.^79^ In treating VAP, it is important to identify or rule out MSSA and MRSA. In a study of BAL samples from 328 patients with VAP, a PCR approach using the Xpert^®^ assay (Cepheid) was shown to rapidly test for MSSA and/or MRSA with high reliability; the negative predictive values (NPVs) for MSSA and MRSA were 99.7% and 99.8%, respectively.^80^

Automated microscopy approaches using techniques such as PNA-FISH and automated phenotypic growth pattern analysis have been successfully used in resistance typing of various pathogens, including *K. pneumoniae*, *E. coli*, *Enterobacter* spp. and other GNBs.^49,81^ RDT typing using the ID/AST system (Accelerate Diagnostics Inc.) reduced turnaround time from 51.4 h to 10.2 h; consequently, antibiotic de-escalation occurred in most patients.^81^ For patients receiving an inactive regimen, the ID/AST method would potentially have allowed appropriate therapy 35.8 h sooner and de-escalation 41.1 h sooner.^81^ Overall, POCTs that include rapid antibiotic resistance typing have the potential to substantially reduce morbidity and mortality in nosocomial pneumonias and various other GNB sepsis cases. These methods can identify resistance mechanisms in 6 h, with reported sensitivity of 89% and specificity of 97%.^36^

BSI represents an increasing public health concern. The estimated incidence of sepsis is 31.5 million per year worldwide, with potentially 5.3 million annual deaths linked to sepsis.^82^ Moreover, in BSI caused by MDR pathogens, for example with carbapenem-resistant *K. pneumoniae*, a pooled mortality of 54.3% has been reported.^83^ Furthermore, delayed effective therapy is associated with worse outcomes.^84^ Delayed identification of the causative organisms and culture susceptibilities may often be responsible for delays in optimal antimicrobial therapy, and this emphasizes the need for rapid identification of antibiotic susceptibility profiles and detection of resistance genes. In BSI, rapid diagnostic testing was associated with significant decreases in mortality risk when combined with an AMS programme, and also decreased the time to effective therapy and hospital LOS.^85^

cUTI is a common infection. The treatment of cUTI is more challenging when it is caused by MDR pathogens, especially ESBL-producing GNB, and inappropriate treatment is associated with clinical failure and mortality. Compared with non-ESBL urinary tract infections (UTIs), ESBL UTIs are associated with prolonged time to appropriate antibiotic use and therefore to prolonged hospital LOS and higher cost of care.^86^ In one study, the use of RDTs was shown to reduce use of ineffective antibiotics to treat UTIs and increase the use of accurate, narrow-spectrum antimicrobials, helping to achieve the goals of AMS.^87^

## Future directions in rapid diagnostic testing

As technologies advance and become more accessible, rapid antibiotic resistance screening methods are likely to become more important components of future diagnostic protocols for typing GNB and other pathogens causing sepsis and other infections, and are likely to be incorporated into AMS initiatives.^88^ A quickly advancing technology is microfluidics, in which liquid broth samples containing bacterial cells are introduced into a channel in a disposable cartridge that divides into multiple parallel channels with over 8000 docking sites.^89^ Growth at these sites is usually recorded microscopically, but electrical resistance sensing is also possible.^90–92^ This technology has been termed ‘lab-on-a-chip’.^40^ Some current devices can screen four different antibiotics simultaneously and provide results in 15–30 min, although others have reported turnaround times up to 3 h.^89,93^ MICs determined using microfluidics have matched those derived from conventional microdilution methods.^89^ Several commercial microfluidic systems are available (e.g. QuickMIC^®^ [Gradientech], Q-linea ASTar^®^, 216Dx^®^ [BacterioScan] and oCelloScope^™^ [BioSense]) and these are likely to become more capable and applicable to a wider range of samples and antibiotic resistance mechanisms in the future. Versions of this technology have been developed to incorporate antibody-coated microbeads to capture specific strains and provide fluorescence detection of antibiotic susceptibility.^94^ Other types have been developed with antibody-coated nanotubes, which may also have applications in resistance detection.^95^

A further advancing technology for resistance typing is MS, particularly MALDI-TOF and, potentially, electrospray ionization in combination with PCR.^96^ MS requires sophisticated and costly hardware and data processing, but it is becoming more available to clinical laboratories at the point of care, and the range of resistances it can detect is increasing. Sample processing in commercial systems is simple, involving spotting cultures directly onto MS target plates with antibiotics, incubation for 3–4 h, followed by MS analysis. MS techniques are likely to play an increasing role in rapid resistance typing in the future.^32,40,43,45^ Some MS techniques also have the advantage that they can be used with raw clinical samples, eliminating delays involved in pathogen culture.

In colorimetric assays for detecting volatile compounds indicative of bacterial growth, future developments are likely to include commercially available microwell plates pre-coated with antibiotics that can be read using smartphone-based devices.^40,97^ This approach is attractive, being low-cost and readily usable close to the point of care.

Rapid molecular methods for detecting bacterial resistance are also likely to continue advancing in the future. In PNA-FISH, the panels available for blood cultures are likely to expand to include multiple antimicrobial resistance genes/markers in mixed populations of bacterial species or strains.^98^ WGS is a valuable technique that provides the most extensive information on existing and emerging antibiotic resistance.^40,99^ Whilst WGS has become significantly simpler, more rapid and accessible in recent years, it requires specialist equipment and software. EUCAST concluded that WGS as an AST tool is still either poor or non-existent and is inadequate for clinical decision-making.^40,100^ The establishment of standardized, internationally agreed analytical approaches and interpretive criteria for WGS, the creation of a single database of all known resistance genes and mutations to support comparisons between different approaches, and expansion of the evidence base for WGS-based AST tools are seen as crucial priorities for WGS in order for it to compete with phenotypic AST.^40,100^

It is not clear which RDT will prevail for antibiotic resistance determination or whether multiple different methods will be used in parallel. Each approach needs more extensive evaluation and comparison before optimal diagnostic pathways emerge and consensus protocols and guidelines can be developed.^96^ Genotypic tests, which incur substantially higher costs, are therefore more likely to be used in a complementary fashion to traditional phenotypic methods.^42^ To become accessible as a POCT, genotypic methods would have to forego expensive devices, laborious sample preparation and high-tech laboratory facilities.^40^ Nevertheless, these rapid methods collectively provide considerable potential for future rapid typing of bacterial resistance and provide greater confidence in treatment selection leading to improved outcomes.

The COVID-19 pandemic caused a large increase in the development, commercialization and approval of SARS-CoV-2 RDTs, with ease of use and rapidity of testing identified as important criteria to reduce the spread of the virus.^101^ A key factor in the expediated implementation of RDTs for COVID-19 was the adaptation of regulatory bodies’ guidelines and policies for approval of diagnostic technologies.^101^ Global partnerships were also developed to allow low- and middle-income countries access to affordable testing; whether these existing partnerships could be exploited for other RDTs is uncertain, but the pandemic has shone a light on RDTs and hopefully paved the way for their further development and more widespread use in other fields, such as clinical bacteriology.^101^

## Considerations when choosing empirical and targeted therapies

Before the availability of novel agents with activity against DTR-GNB, targeted therapy of infections in critically ill patients was mostly based on the administration of polymyxins for DTR-GNB without intrinsic resistance to these agents. This was either as a monotherapy or in combination with other agents such as tigecycline, fosfomycin, high-dose carbapenems and/or sulbactam, with the choice of treatment depending on the type of DTR-GNB and the site of infection. For most scenarios, data from randomized controlled trials (RCTs) are not available to guide empirical therapy in the setting of suspected DTR-GNB infection.^102–105^ In this uncertain situation, the rationale for polymyxin-based combination therapy was mostly based on the possible recognized suboptimal effectiveness of polymyxins used alone and rising *in vitro* polymyxin resistance rates.^105–107^ Notably, as polymyxin monotherapy in the management of DTR-GNB infections (including CRE, CRPA and CRAB) is not recommended,^105^ the dilemma of better polymyxin-based combinations for empirical therapy is still unresolved. However, the AIDA trial^108^ showed that adding meropenem to colistin did not add any benefit, and several less toxic and more effective novel agents may be more suitable. Increased resistance to polymyxins and a lack of clarity on the effectiveness of different combinations increases the importance of understanding how to make best use of novel agents in line with AMS principles. A more detailed selection of both empirical and targeted therapies may now be directed toward specific resistance determinants of specific DTR-GNB. For example, cefiderocol, ceftazidime/avibactam, meropenem/vaborbactam and imipenem/relebactam display activity against KPC-producing CRE, and ceftazidime/avibactam and cefiderocol are also active against OXA-producing CRE.^109–115^ The choice is more limited for MBL-producing CRE, which may be restricted to cefiderocol (although the susceptibility of NDM producers is not universal) or the combination (although evaluated only in observational studies to date) of ceftazidime/avibactam with aztreonam.^116–120^*In vitro* susceptibility studies and pharmacokinetic models are available for this combination against MBL-producing Gram-negative pathogens, but further studies are needed. In the meantime, IDSA recommends this combination for the treatment of MBL (i.e. NDM, VIM or IMP) carbapenemase-identified Enterobacterales.^119,120^ For MDR *P. aeruginosa*, while ceftolozane/tazobactam and ceftazidime/avibactam frequently have comparable *in vitro* activity, ceftolozane/tazobactam does not have activity against CRE.^121,122^ In a retrospective, observational cohort study, ceftolozane/tazobactam was shown to have a higher clinical cure rate when compared with polymyxin or aminoglycoside-based treatment for drug-resistant *P. aeruginosa*; however, there was no difference in in-hospital mortality.^121^ However, in a real-world study where delayed initiation of treatment for MDR *P. aeruginosa* infection was common (therapy started a median of 9 days after culture collection), starting ceftolozane/tazobactam within 4 days of culture collection was associated with survival, clinical success and microbiological cure.^123^ The situation for CRAB is more complex, with cefiderocol certainly remaining a promising agent and already an important option in the absence of alternatives but its use for non-fermenters deserves further investigation; the results of the CREDIBLE RCT showed an unfavourable effect on mortality, which needs to be confirmed in larger studies.^124,125^ On the other hand, it is important to consider that (i) the role of BL/BLI combinations and cefiderocol in increasing probability of coverage and chances of clinical success, and also old and novel agents belonging to classes other than BL/BLIs, would also need to be refined within future therapeutic algorithms; and (ii) at present old agents such as polymyxins still hold a place in the therapy of severe DTR-GNB infections that are resistant to novel agents.

Various studies show that the use of inappropriate antibiotic treatment or delays as short as 24 h for MDR infection leads to treatment failure and poor outcomes. In a real-world study, among 112 patients with identified MDR infections, the antibiotic failure rate was 68.3% and the mortality rate was 40.8%.^9^ This emphasizes the critical importance of selecting the correct initial antibiotic treatment, but also the value of rapid diagnostic methods that can inform treatment decisions at the earliest possible stage.^9,10^ The value of a prompt start to appropriate therapy was emphasized by a study of 102 patients with KPC-Kp BSI.^5^ The median time to appropriate antibiotic therapy in survivors was 8.5 h versus 48 h for those who died (*P *= 0.014) and time to appropriate therapy was an independent predictor of 30 day mortality (HR = 0.36, *P *= 0.0021). This study also identified primary bacteraemia, cardiovascular disease, SOFA score and increasing age as risk factors for 30 day mortality due to KPC-Kp BSI.

AMS will be crucial for preserving the effectiveness of new agents in the long term to assist in the avoidance of indiscriminate use and, at the same time, to guarantee their prompt use in those who may benefit the most from their administration. This may occur through the early identification of patients at risk by accurate syndromic approaches built on patient-level data and on the local microbiological epidemiology. It will also help in selecting the most appropriate empirical therapy. Furthermore, an optimized use of RDT would likely be essential for guiding both targeted therapy with novel agents and rapid de-escalation/discontinuation when they are no longer necessary.

## Conclusions

The treatment of MDR-GNB presents many challenges. Since an effective treatment should be administered as soon as possible, resistance to many antimicrobial classes almost invariably reduces the probability of adequate empirical coverage, with possible unfavourable consequences. Several factors need to be considered to optimize appropriate therapy. One is recognizing the patient-level risk of infections due to DTR-GNB based on medical history and previous colonization or infection with resistant organisms; another is to be informed through updated local epidemiology about the prevalent mechanisms of resistance, to quantify the risk of DTR-GNB infections. In addition, improving and anticipating aetiological diagnosis through phenotypic and molecular resistance typing techniques will help in the selection of the right antibiotic, including the novel BL/BLIs which are active against different types of carbapenemases. Finally, a rapid detection of DTR-GNB will improve targeted therapy through rapid initiation of adequate therapy and de-escalation to a narrow-spectrum antimicrobial when results are available, decreasing the possibility of selective pressure.

As outlined earlier, RDTs have some limitations (e.g. high costs, lack of access in developing countries, resistance coverage); however, POCTs and molecular (genotyping) assays have significant advantages compared with standard culture methods. They have higher sensitivity and specificity and accelerate the detection of MDR organisms to guide directed therapy and infection control practices. This leads to a rapid de-escalation of broad-spectrum antimicrobial agents, reducing treatment costs, spread of MDR pathogens and the potential emergence of future resistance. However, although culture methods for determining antimicrobial resistance in GNB are time-consuming and results may take 24–48 h, delaying appropriate treatment, they remain the gold standard. In response to this clinical need, a range of rapid diagnostic methods for GNB resistance typing are now available and others are under development. The integration of novel and rapid diagnostics for resistance phenotyping in patients has the potential to improve treatments and outcomes of MDR-GNB infections. In addition, understanding the risk factors and epidemiology for Gram-negative MDR infection and a knowledge of pre-clinical and clinical data on new antibiotics, as well as using diagnostic and treatment algorithms, is vital for an appropriate empirical treatment. Finally, early appropriate diagnostics and treatment of MDR Gram-negative infections needs a multidisciplinary approach that includes multiple different diagnostic methods and further consensus of algorithms, protocols and guidelines to select the optimal antibiotic therapy.

## Transparency declarations

The authors declare the following financial interests/personal relationships that may be considered as potential competing interests. M.B. has received research grants and/or has been an advisor/consultant and/or speaker/chairman for the following: Angelini, Bayer, bioMérieux, Cidara, Cipla, Gilead, Menarini, MSD, Pfizer and Shionogi. S.S.K. has received honoraria for advisory boards, conferences from Pfizer, Merck, Gilead, Novartis and Menarini, and honoraria for author contributions to UpToDate. C.R. has received advisory board and speaker honoraria from Pfizer, Sanofi and bioMérieux, and speaker honoraria from B. Braun, Becton Dickinson, Cipla, Glenmark, Novartis and Cepheid. D.v.D. is a consultant for Actavis, Tetraphase, Sanofi-Pasteur, MedImmune, Astellas, Merck, Allergan, T2Biosystems, Roche, Achaogen, Neumedicine, Shionogi, Pfizer, Entasis, QPex, Wellspring, Karius and Utility. D.v.D. receives an editor’s stipend from BSAC. D.v.D. reports grants from Shionogi, Merck and NIH, outside the submitted work. M.V.V. has received honoraria and research grants from MSD, Pfizer, bioMérieux, West and GPC Pharma. All authors received an honorarium from Pfizer to attend a steering committee meeting to discuss their regional perspective on MDR Gram-negative infections and AMS strategies; the output of this discussion was used to create to a framework for this manuscript prior to its development.
